# Breast Density of Mammography is Correlated with Reproductive Risk Factors Regardless of Menopausal Status: A Cross-Sectional Study of the Korean National Screening Program

**DOI:** 10.31557/APJCP.2020.21.4.1011

**Published:** 2020-04

**Authors:** Jung Sun Lee, Minkyung Oh

**Affiliations:** 1 *Department of Surgery, Haeundae Paik Hospital, College of Medicine, Inje University, *; 2 *Department of Pharmacology, Inje University College of Medicine, Clinical Trial Center, Inje University Busan Paik Hospital, Busan, Korea. *

**Keywords:** Breast, screening, mammography, menopause

## Abstract

**Objective:**

To clarify the limitations of mammography screening for women with dense breasts, we examined breast density and its effects on screening results.

**Patients and Methods:**

We performed a cross-sectional, observational study on women who underwent mammography. Data from the National Cancer Screening Program(NCSP) from 2009 to 2013 were used. The study population consisted of participants with high breast density. We used a logistic regression analysis to evaluate the relationships between breast density and reproductive factors and screening results according to menopause status.

**Results:**

High breast density was reported for 57.5% of all participants (3,417,319 participants). Screening results indicated breast density of <25%, 25-50%, 51-75%, and ≥76% for 16.4%, 26.3%, 37.8%, and 19.5%, respectively, of participants. According to the screening results, high breast density was correlated with high deferment and recall rates. Reproductive factors, especially parity, breastfeeding, and use of oral contraceptives, had consistent effects on screening results of premenopausal and postmenopausal women. Regardless of menopausal status, age, early onset of menarche (15 years or younger), fewer live births (≤1 birth), and previous benign breast disease were correlated with increased breast density. In postmenopausal women, early-onset menopause and longer-term hormone replacement therapy (≥2 years) also independently increased breast density.

**Conclusion:**

Breast density influenced screening results, which could increase the rate of recall. Breast density was also influenced by reproductive factors, with patterns similar to those of breast cancer risk, regardless of menopausal status. We need to identify high-risk women with high density who would probably benefit from supplemental breast cancer screening.

## Introduction

Breast density refers to the percentage of dense tissue in the entire breast. Depending on its density, fibroglandular mammary tissue has different characteristics and results in different compositions on radiographs (Pettersson et al., 2014; Patterson et al., 2014). Breast density is an independent predictor of breast cancer risk. Based on studies of Caucasian women living in Western countries, women with more than 75% dense tissue are at four- to six-times higher risk of breast cancer than those without dense breast tissue ( Byrne et al., 1995; McCormack et al., 2006). Although more Asian women than Western women have dense breasts, the incidence of breast cancer is relatively lower among Asian women(Verdial et al., 2017).

Bae et al., (2016) reported an association between breast density on mammography and breast cancer risk for Asian women, regardless of menopausal status. However, Rajaram et al., (2017) suggested that premenopausal Asian and Caucasian women are at similar risk of breast cancer regardless of different breast density. Furthermore, several reproductive and lifestyle factors that influence the risk of breast cancer have been consistently associated with breast density (Brisson et al., 1984; Boyd et al., 2005; Sellers et al., 2007; Martin et al., 2008; Martin et al., 2010).

In Korea, the National Breast Cancer Screening Program (NBSCP) conducts mammography for women older than 40 years biannually. Although the rate of participation has not increased since 2016, the rate of advanced breast cancer detection has not decreased. The false-negative rate of cancer detection by mammography is an unresolved problem; however, the early detection rate of cancer has increased (Suh et al., 2017; Kang et al., 2017). Furthermore, South Korea has a low birth rate. Most women marry at a later age, resulting in lower childbirth rates (Lee et al., 2018).

To maintain the participation of Korean women in breast cancer screening and to improve its efficiency, it is necessary to clarify how factors for Korean could influence the sensitivity of breast screening including breast density. It is also necessary to clarify how breast density affects breast cancer screening and further stratified screening strategies for women at high risk or average- risk with dense breasts. This topic have been discussed in Asian country where the incidence of breast cancer has been rapidly increasing, but large-scaled population based study in these area were needed. Therefore, we conducted a cross-sectional analysis of the Korean National Screening Program from 2009 to 2013 to determine the distribution of breast density and its correlation with reproductive factors and screening results.

## Materials and Methods

*Study population*

This cross-sectional observational study was conducted using data maintained by the NCSP during 2009 to 2013. The NCSP recommends that all Korean women 40 years and older should undergo mammography biannually. 

The baseline cohort comprised 9,469,234 women 40 years and older who underwent screening by the NCSP between 2009 and 2013. We excluded 5907 participants with a previous diagnosis of breast cancer and 6,051,915 participants with unknown breast density according to mammography. Therefore, a total of 3,417,319 Korean women aged 40 years or older were included, and their 7,265,584 available screening results were analyzed.

The current study collected data from the NCSP database, which included information regarding participant demographics and screening results. Written informed consent was received from participants for the collection of their screening results. We collected data regularly from the NHIS. The need for informed consent for this specific study was waived because the NCSP database is quite large. After obtaining permission from the Ministry of Health and Welfare, the investigators used data maintained and de-identified by the NHIS. The study was approved by the Institutional Review Board of Inje University Haeundae Paik Hospital in Korea (IRB no. 2017-07-639-009).

*Categorization of mammographic density and screening results*

The most commonly used tool for assessing breast density is the breast imaging reporting and data system (BI-RADS). Level one indicates a breast comprising almost entirely fatty tissue with 5-24% tissue density. Level two indicates breast tissue comprising scattered areas with 25-49% density and fatty tissue. Level three indicates heterogenous density with 50-75% tissue density and areas of non-dense tissue. Finally, level four indicates ≥75% tissue density with very little to no fatty tissue and is designated as extremely dense. Women with heterogeneously dense or extremely dense breast tissue are considered to have high breast density (Balleyguier et al., 2007).

Screening results were sent to participants and classified into four qualitative categories: normal, benign breast disease, suspected malignancy, and deferred. A percentage of MDMD was not included in the report of screening results. However, the report indicated the limitation caused by breast density and indicated that participants with more than 50% dense breast tissue should revisit the screening center for further examination, even if the results were normal or if they had any breast cancer symptoms such as breast pain, lump, or nipple discharge. Women with suspected malignancy and those who were deferred were also encouraged to undergo further screening. 

*Selected reproductive risk factors*

The NCSP also collected other breast cancer risk factors from the self-reports of participants. Collected data included current history of benign breast disease (no, yes, or unknown), parity (0, 1, or ≥2), duration of breastfeeding (never, <6 months, 6-11 months, ≥12 months), age at menarche (younger than 13, 13-15, 16 or older), duration of oral contraceptive use ( never, <1 years, 1-2 years, unknown), age at menopause (younger than 50, 51-54, 55 or older), and hormonal replacement therapy use (never, <2 years, 2-4 years, ≥5 years, or unknown).

*Statistical analysis*

Because the associations between reproductive risk factors and screening results varied according to the menopausal status, and because PMD was lower for postmenopausal women compared to premenopausal women, all analyses were conducted separately for premenopausal and postmenopausal women (menopausal status was defined at the time of the mammogram). The chi-square test was used to evaluate the correlation between screening results and personal characteristics. Logistic regression was conducted to determine personal risk factors for breast density. Analyses were conducted using SAS version 9.4 (SAS Institute, Cary, NC, USA), and results were considered statistically significant if P ≤ 0.05.

## Results

*Characteristics of participants*

Among 7,265,584 screening results, 16.4%, 26.3%, 37.8%, and 19.5% had level one, level two, level three, and level four breast density, respectively. More than 50% breast density was present in 57.3% of women (Suppl. Table 1).

*Screening results and recall rates according to breast density*

According to the screening results, breast density was inversely correlated with a high frequency of normal or suspected malignancy, and high breast density was correlated with a high frequency of deferment (χ^2^ = 262,313; P < 0.001) (Table 1). Recall cases also increased with higher breast density. For breast density <25% and 49-50%, the recall rates were 104 and 189 per 1000 participants; however, for breast density 50-75% and ≥75%, the recall rates were 290 and 330 per 1000 participants (Table 1).

*Associations between reproductive factors and screening results*

Breast density and reproductive risk factors influenced the screening results, but the patterns of influence were different. Older age decreased the deferment rate instead of increasing the normal and suspected malignancy rates. Nulliparous women tended to be recalled because of a relatively high rate of deferment or suspected malignancy. Longer breastfeeding durations (≥12 months) increased the proportion of participants with normal results and suspected malignancy compared to women who did not breastfeed (Table 2). Parity, duration of breastfeeding, and use of oral contraceptives were consistently correlated with screening results, regardless of the menopausal status (Suppl Table 2).

*Breast density and reproductive factors for premenopausal and postmenopausal women*

For premenopausal participants, younger age at screening was correlated with high breast density. Early onset of menarche (15 years or younger), nulliparity, and absence of previous benign breast disease was correlated with high breast density. Never breastfeeding was correlated with high breast density. The multivariate analysis indicated that all these reproductive factors were independently correlated with breast density (Table 3). For postmenopausal participants, age at screening, age at menarche, parity, breastfeeding, and previous benign breast disease were also correlated with breast density according to the univariate and multivariate analyses. Younger age at menopause was correlated with high breast density according to the univariate analysis but inversely correlated with high breast density according to the multivariate analysis. Hormone replacement therapy (HRT) was inversely correlated with breast density (Table 4). Odds ratios of age at menarche, parity, breastfeeding, and previous benign breast disease were more increased in postmenopausal women than in premenopausal participants.

**Table 1:** Correlation between Breast Density and Screening Results from 2009 to 2013

MD	<25%		25-49%		50-75%		≥76%		Total	
	N	%	N	%	N	%	N	%	N	%
Normal	919,025	77.14	1,234,907	64.68	1,507,732	54.87	756,543	53.39	4,418,207	50.5
Benign breast disease	147,596	12.39	313,786	16.44	442,178	16.09	193,002	13.62	1,096,562	19
Suspected malignancy	7,460	0.63	11,593	0.61	11,679	0.42	4,971	0.35	35,703	0.62
Deferred	117,317	9.85	348,842	18.27	786,455	28.62	462,498	32.64	1,715,112	29.7
case*	124,777		360,435		798,134		467,469		1,750,815	
Recall rate per 1000	104		189		290		330		241	
Total	1,191,398	100	1,909,128	100	2,748,044	100	1,417,014	100	7,265,584	100

*Total number, number of deferred cases + number of suspected malignancy cases

**Table 2:** Correlation between Screening Results and Reproductive Factors

Screening results	Normal		BBD		Suspected Malignancy		Deferred		Total	
Clinical Factors	N	%	N	%	N	%	N	%	N	%
Age (years), χ^2^=65,586, p<0.001										
≥40	1,548,673	59.2	331,695	12.7	9747	0.4	724,723	27.7	2,614,848	100
≥50	1,724,667	60.8	445,787	15.7	13,212	0.5	652,560	23	2,836,230	100
≥60	804,767	61.6	231,044	17.7	8047	0.6	262,722	20.1	1,306,581	100
≥70	359,708	66.3	93,120	17.1	4940	0.9	85,086	15.7	542,854	100
Parity, χ^2^=12,322, p<0.001										
1 child	515,771	57.6	137,479	15.4	4307	0.48	238,208	26.6	895,768	100
≥2 children	3,700,165	61.7	897,365	14.9	29,206	0.49	1,372,633	22.9	5,999,376	100
None	197,988	55.2	58,486	16.3	2280	0.64	100,197	27.9	358,951	100
Breastfeeding, χ^2^=13,524, p<0.001										
< 6 months	739,747	59.8	178,534	14.4	4852	0.39	314,418	25.4	1,237,551	100
≥6 but <12 months	955,465	60.2	238,840	15.1	6760	0.43	384,885	24.3	1,585,956	100
≥12 months	2,054,368	62.6	499,011	15.2	18,526	0.56	709,972	21.6	3,281,880	100
Never	566,726	58.4	148,465	15.3	4547	0.47	250,742	25.8	970,481	100
Previous BBD, χ^2^=19,450, p<0.001										
Yes	635,649	56.3	209,110	18.5	5090	0.45	279,965	24.7	1,129,815	100
None	3,523,740	61.9	812,179	14.3	27,627	0.49	1,322,159	23.3	5,685,712	100
Unknown	256,725	57.7	73,700	16.5	3098	0.7	111,592	25.1	445,117	100
Menstruation, χ^2^=52969, p<0.001										
Yes	1,682,997	38.1	373,342	34.1	11,548	32.3	796,126	46.5		
Hysterectomy	405,609	9.2	102,828	9.4	2696	7.5	138,874	8.1		
Menopause	2,326,662	52.7	618,654	56.5	21,562	60.2	778,619	45.4		
Total	4415268	100	1094824	100	35803	100	1713619	100		
Oral contraceptive, χ^2^=1017.2, p<0.001										
Never	3,571,943	80.9	875,827	80.2	29,246	81.7	1,388,139	81.1		
<1 year	419,988	9.5	105,457	9.6	3134	8.8	164,804	9.7		
≥1 but <2 years	224,420	5.1	58,746	5.4	1742	4.8	80,938	4.7		
Unknown	195,398	4.4	52,529	4.8	1667	4.7	77,019	4.5		
Total	4411749	100	1092559	100	35789	100	1710900	100		
HRT, χ^2^=2799.4, p<0.001										
Never	1,690,208	72.8	453,590	73.5	17,687	82.2	579,290	74.64		
<2 years	29,297	12.6	73,933	11.9	1792	8.3	93,094	11.9		
2-5 years	137,511	5.9	34,582	5.6	658	3	40,609	5.2		
≥5 years	113,536	4.8	29,804	4.8	540	2.5	32,554	4.2		
Unknown	87,455	3.7	24,924	4	848	3.9	30,592	3.9		
Total	2,058,007	100	616,833	100	21,525	100	776,139	100		

**Table 3:** Univariate and Multivariate Analyses of Personal Characteristics of Premenopausal Women and Mammography Density

		Univariate				Multivariate			
		OR	95% CI		P value	OR	95% CI		*P*-value
Premenopausal	Age (years)				<0.001				<0.001
	40-49	14.82	7.87	27.88		16.69	8.85	31.47	
	50-59	3.32	1.71	6.45		3.58	1.84	6.98	
		1	ref			1	ref		
	Menarche (years)				<0.0001				<0.0001
	<13	1.12	1.06	1.18		1.04	0.98	1.1	
	13-15	1.23	1.2	1.27		1.14	1.1	1.17	
	≥16	1	ref			1	ref		
	Parity				<0.0001				<0.0001
	Ever	1.63	1.51	1.76		1.43	1.31	1.56	
	Never	1	ref			1	ref		
	Breastfeeding				<0.0001				<0.0001
	Ever	1	1.25	1.34		1.14	1.09	1.18	
	Never	1.29	ref			1	ref		
	Previous BBD				<0.0001				<0.0001
	Yes	1.37	1.3	1.43		1.43	1.36	1.49	
	None	1	ref			1	ref		
	Oral contraceptives				0.0004				0.0009
	Never	1	1.03	1.12		1.07	1.03	1.11	
	Ever	1.07	ref			1	ref		

**Table 4:** Univariate and Multivariate Analyses of Personal Characteristics of Postmenopausal Women and Mammography Density

		Univariate				Multivariate			
		OR	95% CI		*P* value	OR	95% CI		*P* value
Postmenopausal	Age (year)				<0.0001				<0.0001
	40-49	15.92	14.7	17.25		14.69	13.52	15.97	
	50-59	8.39	7.84	8.97		7.47	6.98	7.99	
	60-60	3.06	2.85	3.28		2.80	2.61	3.01	
	≥70	1.00	ref			1.00	ref		
	Menarche (year)				<0.0001				<0.0001
	<13	1.26	1.18	1.35		1.12	1.03	1.2	
	13-15	1.56	1.52	1.61		1.21	1.17	1.24	
	≥16	1.00	ref			1.00	ref		
	Parity				<0.0001				<0.0001
	Ever	1.9	1.77	2.04		1.39	1.28	1.52	
	Never	1.00	ref			1.00	ref		
	Breastfeeding				<0.0001				<0.0001
	Ever	1.00	1.91	2.09		1.42	1.35	1.5	
	Never	1.99	ref			1.00	ref		
	Previous BBD				<0.0001				<0.0001
	Yes	1.96	1.86	2.06		1.59	1.51	1.67	
	None	1.00	ref			1.00	ref		
	Oral contraceptives				<0.0001				<0.0001
	Never	1.00	1.04	1.12		1.09	1.05	1.14	
	Ever	1.08	ref			1.00	ref		
	Menopause (age)				<0.0001				<0.0001
	<50	1.45	1.38	1.53		0.85	0.8	0.89	
	51-54	1.31	1.25	1.38		0.96	0.91	1.01	
	≥55	1.00	ref			1.00	ref		
	HRT (years)				<0.001				<0.0001
	Never	0.65	0.63	0.68		0.68	0.65	0.71	
	<2 years	0.92	0.87	0.97		0.78	0.74	0.83	
	2-5 years	1.00	ref			1.00	ref		

## Discussion

During our retrospective observational study performed at NCSP, we found that breast density influenced screening results and could increase the recall rate. Breast density was also influenced by reproductive factors, with patterns similar to those of breast cancer risk. This finding was consistent regardless of menopausal status.

Parity was significantly and inversely associated with the percentage of collagen in the breast. Smaller breast size was reported to be associated with more collagen and glandular tissue (Li et al., 2005; Rice et al., 2016).

HRT, such as combination estrogen and progesterone, is known to increase breast density, but estrogen therapy alone does not significantly increase breast density(Greendale et al.,2003). Previous reports have found a positive correlation between breast density and HRT that resembles the well-studied relationship between HRT and breast cancer risk (Titus-Ernstoff et al., 2006; Chen et al., 2010).

In interventional trials, breast density has been proposed as a potential surrogate marker of breast cancer risk (Boyd et al., 2011). However, the extent to which reproductive factors influence breast cancer risk through their effects on breast density and the extent to which they influence breast cancer risk through other pathways are unknown (Rice et al., 2016). Additionally, the underlying mechanisms of the positive association between breast density and the risk of breast cancer remain to be elucidated.

Racial differences have been considered an important factor when determining breast density. Asian women tend to have higher breast density and African American women tend to have lower breast density than Caucasian women; furthermore, breast density was found to be significantly higher in Chinese women (Ursin et al., 2003; del Carmen et al., 2007; Heller et al., 2018).

Supplemental imaging modalities have been used to improve examination sensitivity because they can detect breast cancer at an early stage on the basis of the mass shape, even in the dense parenchyma of premenopausal women. Some observational studies have questioned which supplemental ultrasonography method can increase the frequency of interval cancers in women with dense breasts (Kolb et al., 2002; Nothacker et al., 2009; Corsetti et al., 2011; Scheel et al., 2015). Two randomized clinical trials using supplemental imaging have demonstrated a significant reduction in interval breast cancer rates. The Japan Strategic Anti-cancer Randomized Trial (J-START) investigated the efficacy of adjunctive ultrasonography, thereby contributing to the understanding of the efficacy of adjunctive ultrasonography for breast cancer screening of women 40 to 49 years by reporting that adjunctive ultrasonography increases the sensitivity and early detection rates of cancers(Ohuchi et al., 2016). The Dense Tissue and Early Breast Neoplasm Screening Trial reported fewer cases of interval breast cancer with biennial supplemental magnetic resonance imaging plus mammography than with mammography alone for women 50 to 74 years of age with extremely dense breasts (Bakker et al., 2019). Studies have not evaluated whether supplemental imaging reduces advanced breast cancer rates or breast cancer mortality. 

Another modality, digital breast tomosynthesis (DBT), reduced the recall rate significantly for women with dense breasts, indicating that all women may benefit from improved screening with DBT regardless of age or breast density (Conant et al., 2016). After implementation of DBT for screening with digital mammography, the invasive cancer detection rate increased from 2.9 to 4.1 per 1,000 participants, a relative increase of 41%, whereas the detection rate of in situ cancer was unchanged for 1.4 per 1,000 participants(Friedewald et al., 2014).

Melniknow et al. identified one randomized controlled trial that compared the potential danger of different breast density notifications for a control group; however, they reported that there have been no studies of the potential danger of assigning different breast density classifications to sequential examinations (Bottorff et al.,2007; Melnikow et al., 2016). The dangers of supplemental imaging for women with dense breasts include higher recall rates and higher biopsy rates. Long-term follow-up is needed to assess whether a combined approach could reduce the frequency of advanced breast cancers and breast cancer mortality. This approach has not been conducted in Korea, and most participants ignored the screening results because of low sensitivity or had higher recall rates and biopsy rates.

Nelson et al., (2012) found that the only risk factor to confer a two-fold higher relative risk among women 40 to 49 years old was family history in a first-degree relative or extremely dense breasts. Also, they have proposed risk-based screening, such as selective screening of women 40 to 49 years old who are at increased risk of breast cancer, as a means of improving the benefit-to-risk ratio using a meta-analysis. However, a recent retrospective study suggested that 88% and 86% of women with screening-detected breast cancer did not have a very strong family history of breast cancer or extremely dense breast tissue, respectively (Price et al., 2015). Among patients with screening-detected malignancies, 76% did not have a very strong family history or extremely dense breasts (Ray et al., 2018). Kerlikowske et al., (2019) reported the dangers of frequent screening and supplemental imaging and suggested that women aged 50 to 74 years undergoing screening mammography should be informed of their clinical automated BI-RADS breast density and their overall breast cancer risk to determine whether they have dense breasts and are at low risk of breast cancer so they can consider routine biennial screening. Conversely, women with dense breasts and those at high risk of breast cancer due to strong risk factors or those with dense or non-dense breasts and very strong risk factors may want to consider annual screening and supplemental imaging.

In Korea, because of the lower cost of ultrasonography, widespread use of private insurance, and confidence in ultrasonography, most women tend to supplement breast examinations with ultrasonography. Supplemental testing could consistently find additional breast cancers not identified by mammography, but it could also increase false-positive results. Additionally, no studies have examined the impact of supplemental screening on breast cancer recurrence rates or mortality for women with dense breasts in Korea.

The present study is the first report about MD with breast screening mammography from NCSP data. Almost 57.3% of Korean women had more than 50% breast density by mammography. Because a high rate of high breast density was found in the NCSP, there occurred two major issues. First, high breast density decreases the detection sensitivity of mammography screening and increases the recall rate in NCSP. Second, breast density was probably known as an independent risk factor for breast cancer, and it needs to evaluate who should be notified about their MD. In Korea, high breast density is an issue when attempting to detect breast cancer, it has started to be known as an independent risk factor of breast cancer for these women from several studies (Kim et al., 2015; Park et al., 2018). Furthermore, when adjusted for age according to unpublished data, breast density was found to have a significant effect on breast screening results and the breast cancer diagnosis. However, we did not disclose these data because data from the NHIS were categorized and not continuous; therefore, they could not be verified.

In conclusion, high breast density was found in 57.5% of the study participants. Breast density influenced the screening results, which could lead to increased recall rates. Breast density was also influenced by reproductive factors, with patterns similar to those of breast cancer risk, regardless of menopausal status. It is possible that breast density could be a limitation of breast cancer screening in Korea. We need to identify high-risk women who would probably benefit from supplemental breast cancer screening using population-based screening cohort. Furthermore, women with both normal mammography results and dense breasts need to be discussed to undergo supplemental testing according to their risk of breast cancer in a clinical practice.
